# Frequency of obesity and related risk factors among school children and adolescents in a low-income community. A cross-sectional study: Frequência de obesidade e fatores de risco relacionados em escolares e adolescentes em uma comunidade de baixa renda. Um estudo transversal

**DOI:** 10.1590/1516-3180.2014.8960412

**Published:** 2014-11-28

**Authors:** Mariana Carvalheiro Cotrim Lima, Ceres Concilio Romaldini, João Hamilton Romaldini

**Affiliations:** I Master’s Student, Postgraduate Health Science Program, Hospital do Servidor Público Estadual (HSPE), Instituto de Assistência Médica ao Servidor Público Estadual (IAMSPE), São Paulo, Brazil.; II MD, PhD. Professor, Postgraduate Health Science Program, Hospital do Servidor Público Estadual (HSPE), Instituto de Assistência Médica ao Servidor Público Estadual (IAMSPE), São Paulo, Brazil.; III MD, PhD. Titular Professor of Endocrinology, Pontifícia Universidade Católica de Campinas (PUC-Campinas), and Professor, Postgraduate Health Science Program, Hospital do Servidor Público Estadual (HSPE), Instituto de Assistência Médica ao Servidor Público Estadual (IAMSPE), São Paulo, Brazil.

**Keywords:** Obesity, Risk factors, Nutritional status, Cardiovascular diseases, Lipids

## Abstract

**CONTEXT AND OBJECTIVE:**

The frequency of obesity at an early age may contribute to atherosclerosis and cardiovascular disease (CVD) in adults. This study measured the frequency of obesity and cardiovascular risk factors in children and adolescents aged 6 to 17 years.

**DESIGN AND SETTING:**

Cross-sectional study in a school located in a region of low income and socioeconomic status in Santa Rita do Sapucai, Minas Gerais, Brazil.

**METHODS:**

A total of 175 students were classified using body mass index (BMI) and their waist circumference, blood pressure, number of hours of sedentary behavior and school meals were evaluated. Serum concentrations of fasting blood glucose, total cholesterol (TC), triglycerides (TG), low-density lipoprotein (LDL-C) and high-density lipoprotein (HDL-C) were analyzed.

**RESULTS:**

37.2% of the students had BMI above the 85^th^ percentile and had significantly lower age, higher prevalence of hypertension, higher serum TC, LDL-C and TG, and greater waist circumference than those with BMI below the 85^th^ percentile. Hypertension was observed in 2.9% of the students; 5.1% presented impaired glucose tolerance, 40% had two risk factors for atherosclerosis and 26.9% had three risk factors. A sedentary lifestyle was significantly less prevalent among subjects with BMI above the 85^th^ percentile and was significantly correlated with serum TC and LDL-C. The school meals were hypoglycemic, hyperproteic and hyperlipidemic.

**CONCLUSION:**

One third of the children and adolescents had weights greater than or equal to the age-adjusted weight, and this was associated with greater waist circumference, hypertension and prevalence of dyslipidemia.

## RESUMO

**CONTEXTO E OBJETIVO:**

A frequência de obesidade em idade precoce pode contribuir para a aterosclerose e doença cardiovascular (DCV) em adultos. Este estudo mediu a frequência de obesidade e fatores de risco cardiovascular em crianças e adolescentes com idades entre 6 e 17 anos.

**TIPO DE ESTUDO E LOCAL:**

Estudo transversal em escola localizada numa região de baixa renda e baixo nível socioeconômico em Santa Rita do Sapucaí, Minas Gerais, Brasil.

**MÉTODOS:**

Um total de 175 alunos foi classificado pelo índice de massa corporal (IMC) e foram aferidas circunferência da cintura, pressão arterial, horas de sedentarismo e alimentação escolar. Realizaram-se dosagens séricas de glicemia de jejum, colesterol total (CT), triglicerídeos (TG), lipoproteína de baixa densidade (LDL-C) e lipoproteína de alta densidade (HDL-C).

**RESULTADOS:**

37,2% dos alunos tinham IMC acima do percentil 85 e apresentaram idade significativamente menor, prevalência mais alta de hipertensão, CT sérico, LDL-C e TG elevados e maior circunferência abdominal em comparação com aqueles com IMC abaixo do percentil 85. Observou-se hipertensão em 2,9% dos estudantes, 5,1% apresentavam tolerância à glicose diminuída, 40% tinham dois fatores de risco para aterosclerose, e 26,9% tinham três fatores de risco. O sedentarismo foi significativamente menor em indivíduos com IMC acima do percentil 85 e foi significativamente correlacionado com as concentrações séricas de CT e LDL-C. A merenda escolar estava hipoglicêmica, hiperproteica e hiperlipídica.

**CONCLUSÃO:**

Um terço das crianças e adolescentes avaliados tinham pesos iguais ou superiores ao peso ajustado à idade, e isso foi associado a maiores circunferência abdominal, hipertensão e prevalência de dislipidemia.

## INTRODUCTION

Cardiovascular disease (CVD) is preceded by atherosclerosis, which is characterized by formation of plaque containing calcified necrotic nuclei, accumulation of lipids and leukocytes, and smooth muscle inflammation.^1^ This plaque develops through atheromatous deposits within arterioles and arteries that appear early in life, primarily in overweight children and adolescents.^2^ Approximately one third of all deaths worldwide and 85% of deaths in low and middle-income populations are attributed to CVD.^3^ In Brazil, similar to the situation in other developing countries, there are 300 deaths for CVD for every 100,000 inhabitants,^4^ and 30% of these deaths are related to atherosclerosis.^5^^,^^6^ Some patients may have one or more CVD risk factors, such as hypertension, diabetes mellitus, hyperinsulinemia, smoking, dyslipidemia or low physical inactivity. However, all of these factors can be prevented during childhood and adolescence.

In a study on 109 children and adolescents with a family history of premature CVD, 38.5% of the subjects had dyslipidemia alone or in association with atherosclerosis risk factors such as physical inactivity, hypertension, obesity and smoking. Overweight children and adolescents had a 2.8-times greater risk of developing dyslipidemia, and 72.5% of the patients affected did not exercise.^7^ Another cohort study showed that weight change during a 10-year follow-up correlated with physical activity. Lower activity was associated with greater gains in body weight, and increasing weight induced a further decrease in physical activity, thus forming a vicious circle.^8^ Furthermore, a study conducted in the United States noted that childhood measurements of serum lipoprotein, hypertension and body mass index (BMI) predicted carotid artery intima-media thickness in young adults.^9^ Collectively, these data suggest that the presence of CVD risk factors in childhood and adolescence may contribute to atherosclerosis development in adults.

The frequency of obesity in Brazil has increased over the last 30 years.^10^ This raises great concern because this increase has been observed from a very early age and at all socioeconomic levels.^11^^,^^12^ Thus, early diagnosing of obesity is important for establishing preventive measures^13^^,^^14^ that could avoid later development of long-term comorbidities.^15^

## OBJECTIVE

The purpose of the present study was first, to evaluate the frequency of overweight and obesity among children and adolescent students in a region of low socioeconomic status; and second, to investigate the possible CVD risk factors (obesity, dyslipidemia, hypertension, large abdominal circumference, high blood glucose and physical inactivity) associated with the nutritional status determined from the BMI.

## METHODS

This cross-sectional study was conducted in a municipal school in the urban area of Santa Rita do Sapucaí, Minas Gerais, which has a population of 37,754 inhabitants.^16^ The participating students were aged between six and 17 years, and the parents or guardians of the children provided informed consent. The exclusion criteria included presence of hypothyroidism, nephrotic syndrome, chronic renal failure or liver diseases and use of corticosteroids, beta-blockers or anabolic steroids.^17^

The study was approved by the Research Ethics Committee of our institution, Instituto de Assistência Médica ao Servidor Público Estadual (IAMSPE). All parents or legal guardians of the participants provided informed written consent prior to inclusion of students in the study.

Family income data were obtained at the time when the students were examined. The maximum income was one minimum monthly wage per capita (equivalent to US$ 308), thus constituting low income and socioeconomic status. A questionnaire on their family story status was completed by one of the authors (MCCL) including information about CVD, diabetes mellitus and hypertension.

A thorough family history was taken by two of the authors (MCCL and CCR), focusing on coronary artery disease, diabetes mellitus and hypertension, along with the child’s history of physical activity and number of hours of sedentary behavior. Time spent on sedentary activities was defined as the number of hours per day spent on activities that do not involve participation in physical activity, including watching television, playing video games, using the computer or no activity.^18^

Body weight was measured on a digital scale with a precision of 0.100 kg. Height was measured by means of a stadiometer, in accordance with Bolzan et al.^19^ BMI was determined using the WHO AnthroPlus software, version 3.2.2, in accordance with the World Health Organization (WHO) reference standard.^20^ The results were expressed as percentiles (P): severely wasted (P < 0.1), wasted (P > 0.1 and P < 0.3), eutrophic (P ≥ 3 and P < 85), overweight (P ≥ 85 and P < 97), obese (P ≥ 97 and P < 99.9) and severely obese (P ≥ 99.9).

Waist circumference was measured at the midpoint between the lower rib and the iliac crest using a nonelastic flexible tape measure, with the subject in a standing position and the waist unclothed.^21^ Abdominal obesity was diagnosed when the circumference was greater than or equal to the 90^th^ percentile.^22^

Measurements of blood pressure were adjusted for age, height and gender and were made with the subject in a seated position after a five-minute rest.^23^ The systolic blood pressure (SBP) and diastolic blood pressure (DBP) were categorized into four percentiles for children and adolescents as follows: normal (< 90^th^ percentile); prehypertension (SBP and/or DBP between the 90^th^ and 95^th^ percentiles); hypertension stage 1 (SBP and/or DBP between the 95^th^ and 99^th^ percentiles); and hypertension stage 2 (SBP and/or DBP ≥ 99^th^ percentile).^23^

The nutritional quality of the school meals was analyzed on three alternate days by one of the authors (MCCL), in relation to the standards of the National School Meals Program (Programa Nacional de Alimentação Escolar, PNAE) and the Brazilian Institute for Geography and Statistics (Instituto Brasileiro de Geografia e Estatística, IBGE).^24^

Peripheral blood samples were collected after overnight fasting by a nurse in vacuette tubes, serum was separated and the biochemical analysis was performed in the same day at the clinical laboratory (Miranda Reis, Santa Rita do Sapucaí, Minas Gerais). The serum concentrations of glucose, total cholesterol (TC), triglycerides (TG), low-density lipoprotein (LDL-C), very low-density lipoprotein (VLDL-C) and high-density lipoprotein (HDL-C) were measured from blood samples taken after 12 hours of fasting. TC and HDL-C were measured by means of an enzymatic colorimetric cholesterol esterase method; TG was also measured using an enzymatic colorimetric method. LDL-C was calculated using the Friedewald formula. Diabetes was defined as fasting glucose greater than or equal to 126 mg/dl; fasting glucose between 100 and 125 mg/dl was defined as impaired glucose tolerance.^25^ The serum lipid reference values followed the first Brazilian guidelines for prevention of atherosclerosis in childhood and adolescence: TC < 170 mg/dl; TG < 130 mg/dl; LDL-C < 130 mg/dl; and HDL-C > 45 mg/dl.^26^

All the participants were informed of the test results and were referred for outpatient care when indicated.

Descriptive analyses were stratified according to gender and BMI (≥ 85^th^ percentile and ≤ 85^th^ percentile). In the bivariate analysis, multiple comparisons were performed using the t test and chi-square test. Multiple comparisons were analyzed using the analysis of variance (ANOVA) test. After confirming a Gaussian sample distribution, linear regression analysis (Pearson correlation) was performed. Significance was designated at 5% for all analyses. The GraphPad Prism 5.01 for Windows software was used for all these analyses (GraphPad Software, Inc., CA, USA).

## RESULTS

Out of 309 students invited, 175 students and their parents (56.6%) agreed to participate. The reminder 134 (43.3%) declined to participate or we were not able to obtain the signed consent. A total 175 children and adolescents were enrolled, comprising 107 females (61.1%) and 68 males (38.9%). Their ages ranged from 5.9 to 17.4 years with a mean age of 11.9 ± 2.1 years (± standard deviation, SD). When the subjects were stratified according to gender and state of puberty, we did not find any difference in the BMI (Fisher exact test, P = 0.06), but the percentage of males with BMI > 85^th^ percentile (42.65%) was significantly higher (Fisher exact test, P = 0.011) than the percentage of females (23.36%).

CVD, diabetes and hypertension were observed in 48%, 49.6% and 62.4% of the participants, respectively. High blood pressure (stages I and II) was observed in 2.9% of the students; 5.1% met the criteria for impaired glucose tolerance, but no cases of diabetes mellitus were diagnosed. Dyslipidemia was found in 6.3% of the subjects with high TC; 2.3% with elevated LDL-C; 1.1% with elevated TG; and 89.7% with HDL-C less than 45 mg/dl. BMI above the 85^th^ percentile was found in 37.2% of the students. Furthermore, 7.1% presented at least one atherosclerosis risk factor, and two, three, four and five risk factors were found in 40%, 26.9%, 12.6% and 3.4% of the subjects, respectively. A total of 61.1% of the subjects had normal weight; 15.4% were overweight, while 16.0% were obese and 4.6% were severely obese. Only 0.6% of the subjects were classified as severely wasted and 2.3% as wasted.

As summarized in Table 1, students with BMI greater than or equal to the 85^th^ percentile had significantly lower age, hypertension, elevated serum TC, LDL-C and TG, and greater waist circumference than shown by individuals with BMI less than the 85^th^ percentile. However, a sedentary lifestyle was observed significantly less frequently among subjects with BMI greater than or equal to the 85^th^ percentile, and the serum HDL-C and glucose levels were not different between the two groups.

**Table 1.**
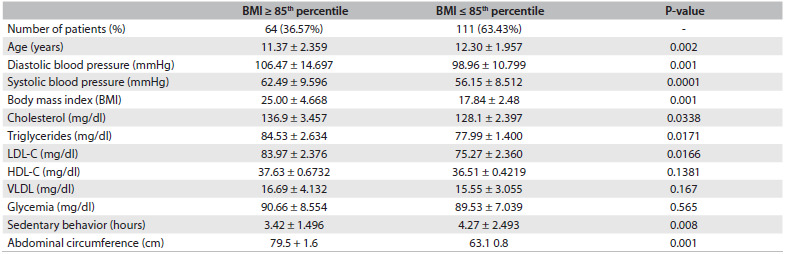
Clinical and laboratory results of the two groups of individuals separated according to body mass index (BMI)

LDL-C = low-density lipoprotein-cholesterol; HDL-C = high-density lipoprotein-cholesterol; VLDL = very low-density lipoprotein.

In subjects with BMI greater than or equal to the 85^th^ percentile, the serum TC and LDL-C levels were significantly higher than those observed in subjects with BMI less than the 85^th^ percentile, as summarized in Table 2.

**Table 2.**
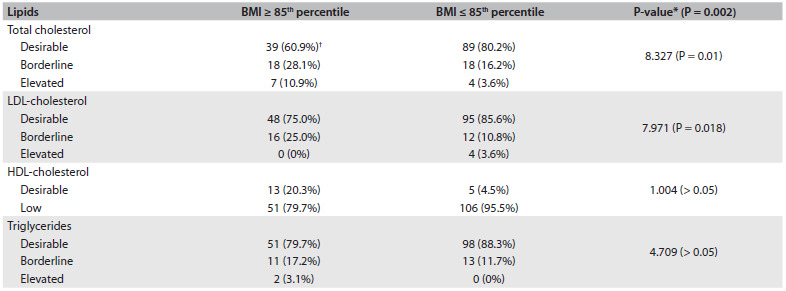
Lipid distribution according to body mass index (BMI) group

*Chi-square test; ^†^Number of subjects and percentages in parentheses. BMI = body mass index; LDL = low-density lipoprotein; HDL = high-density

In males who had reached puberty and whose BMI was greater than or equal to the 85^th^ percentile, we found that the TC levels were significantly higher (21.05%) than in males who had reached puberty and whose BMI was less than the 85^th^ percentile (2.78%; chi-square test, P = 0.028).

In all the subjects, the serum TC and LDL-C concentrations were significantly correlated with the amount of time spent doing sedentary activities (Table 3).

**Table 3.**
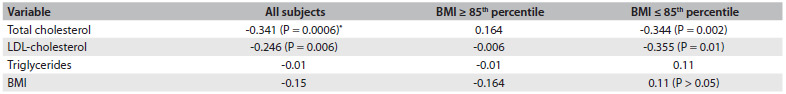
Correlations between history of time spent doing sedentary activities (measured in hours) and some clinical laboratory parameters

LDL = low-density lipoprotein; BMI = body mass index; ^*^Pearson correlation.

The school meals were primarily hypoglycemic (53.1%, rather than the recommended 63%), hyperproteinemic (15.6%, rather than the recommended 12.5%), and hyperlipidemic (31.3%, rather than the recommended 22.5% maximum). Furthermore, while the calcium concentration (72.6%) and magnesium concentration (82.0%) did not reach the daily recommendations,^24^ the iron (103%), zinc (233%), sodium (526.1 mg), vitamin A (178%) and vitamin C (201%) concentrations met the nutritional recommendation and did not exceed the UL (maximum recommended dose).^27^ The dietary saturated fat level exceeded 2.8% of the nutritional recommendation.

## DISCUSSION

The increase in the proportion of overweight children and adolescents over recent decades is an indication of comorbidities relating to obesity in adulthood.^10^ In the present study, 36% of the subjects were overweight, and 48% had a family history of risk factors for CVD and atherosclerosis.^8^^,^^22^ Several studies have confirmed that excess weight is a risk factor for CVD.^8^^,^^22^^,^^23^ Results similar to those of the present study were observed in a study on 3063 children and adolescents, which found that 38.4% were overweight and obese^28^ and that this correlated with increased levels of metabolic diseases and CVD at an early age.^29^

There is a clear correlation between changes in lipoproteins and obesity, and with the onset and severity of childhood atherosclerosis.^30^ The present data were similar to those described by Rover^31^ and Costa,^32^ except for the HDL-C results. The mean TC found in the study by Scherr^33^ was similar to that of the present study. Another study revealed that there were significant differences in all serum lipid parameters between eutrophic, overweight and obese children and adolescents.^28^

The frequency of higher BMI in males than in females may be associated with early sexual maturation.^13^ Overweight explained the elevated TC levels in males who had reached puberty and whose BMI was greater than or equal to the 85^th^ percentile.^28^^,^^31^

We found that students with a BMI greater than or equal to the 85^th^ percentile had significantly elevated DBP and SBP. This was recently also observed in a study on overweight and obese children,^33^ and has been correlated with a fourfold greater risk of hypertension in adulthood.^23^ During physical activity, the adrenergic system becomes more stable and, as a result, serum TG, LDL-C and VLDL-C concentrations decrease while HDL-C increases.^28^^,^^34^ Physical activity also increases cardiac muscle oxygen consumption and improves peripheral microvascular perfusion, thereby preventing atherosclerosis.^34^ The correlation between sedentary behavior and serum TC and LDL-C levels observed in the students with BMI greater than the 85^th^ percentile suggests that increased physical activity has a positive effect on these parameters and on weight.^28^^,^^35^^,^^36^^,^^37^^,^^38^^,^^39^

The nutritional quality of school meals may also negatively contribute to the present findings because of their high fat content, which may gradually increase the serum lipid profile in these students. On the other hand, we did not have access to data on family meals.

## CONCLUSIONS

One third of the children and adolescents living in this low-income and low socioeconomic status community were at or above the recommended age-adjusted weight, which was associated with greater waist circumference, hypertension and dyslipidemia. In addition to dietary change, a decrease in sedentary behavior should be encouraged. These interventions should be implemented at an early age in order to avoid obesity and sedentary behavior later on, thereby decreasing the likelihood of atherosclerosis and CVD in adulthood.
